# The Role of Ceramics in the Configuration of a New Solar Thermal Collection System for Domestic Hot Water and Heating

**DOI:** 10.3390/ma18091907

**Published:** 2025-04-23

**Authors:** Jordi Roviras Miñana, Vicente Sarrablo Moreno

**Affiliations:** Department of Architecture, Universitat Internacional de Catalunya (UIC), Carrer Iradier 22, 08017 Barcelona, Spain; sarrablo@uic.es

**Keywords:** thermal solar collectors, ceramic materials, architectural integration, sustainability

## Abstract

The work presented in this study aims to demonstrate the capacity of ceramic materials in the configuration of solar thermal collectors (CSTs) for the production of domestic hot water (DHW) and heating in buildings. Currently, the ceramic tile and panel manufacturing sector presents very advanced manufacturing systems at a technological level that allows the generation of pieces with high physical and mechanical performances. Especially, their high resistances to extreme temperatures and good thermal conductivities position these materials as great candidates in the field of CSTs. In addition, ceramic materials tend to be durable and corrosion resistant, which makes them a very reliable option in the long term. The results obtained in the test campaign and presented in the article confirm the capacity of ceramics to meet the basic requirements of a CST system for buildings in terms of absorption, energy performance, watertightness, and resistance to water pressure, among other aspects, and make it possible to advance new research to improve the behaviors, performances, and architectural integration of ceramic collectors.

## 1. Introduction

The current geopolitical era is dominated by the climate emergency and requires a commitment to reduce carbon dioxide emissions into the atmosphere, representing a turning point in the construction sector. In developing countries, water heating accounts for 30 to 40% of a household’s energy consumption, mostly produced by gas, fossil fuels, or electricity [1], and, according to the International Energy Agency (IEA), is responsible for 26% of the global energy-related emissions [2]. In fact, buildings currently consume 42% of the world’s electricity and account for 36% of the CO_2_ emissions [3]. Additionally, building materials generate environmental pollution and contribute to global warming through the emission of greenhouse gases, and the availability of building materials is limited, as they are non-renewable resources and susceptible to instability.

Both natural and technological solutions are essential to achieve the COP28 [4] objectives of the transition toward the progressive abandonment of fossil fuels [5]. Given the significant CO_2_ emissions generated by the construction sector, a transformation toward a zero-net-emission building stock is urgently needed.

Nowadays, new construction systems are available that favor the airtightness and thermal insulation capacity of buildings in order to minimize the primary energy consumption and optimize costs, giving rise to the already known Passivhaus (PH) standard, which is mainly based on reductions in energy demand and consumption. In Spain, this passive house concept has become the preferred alternative to achieve nearly zero-energy-consumption buildings (NZEBs) [6]. Directive 2010/31/EU on the energy performance of buildings [7] introduced this NZEB concept in order to promote more efficient buildings, although previously, in December 2002, the European parliament itself approved the Energy Performance Building Directive, EPBD 2002/91/EC [8], with the clear objectives of improving and promoting improvements in the energy performance of buildings throughout Europe, including residential, commercial, and public buildings [9,10]. Even so, in many of the southern European countries, they are not adequately prepared for the correct and effective implementation of such a concept, with many improvements remaining to be addressed in the coming years [11]. Here in Spain, the necessary NZEB criteria were incorporated into the 2019 Technical Building Code (CTE) [12], while the 2020 National Plan [13] incorporates more targeted and focused actions to achieve an energy-efficient and carbon-free building stock by 2050 [14].

In the building sector, in order to achieve high levels of energy efficiency and near-zero energy consumption, it is necessary to introduce renewable energy systems within a building’s own construction system [15], and solar energy is one of the most prominent forms of energy because it is a clean and inexhaustible energy. Moreover, in recent years, solar thermal energy has been the most popular renewable resource used in energy conversion systems, with solar thermal (ST), photovoltaic (PV), and hybrid photovoltaic/thermal (PV/T) systems being the most widely used [16,17,18,19]. According to data from the latest *Solar Heat Worldwide 2024* report generated by the IEA, the global solar thermal capacity of water collectors applied to buildings (houses, offices, sport centers, etc.) increased from 62 GWth (89 million m^2^) in 2000 to 560 GWth (800 million m^2^) in 2023 [20,21]. It is evident, then, that new regulations and directives point to significant growth in the application of such technology in the building field [22].

Even so, a set of limitations and contradictory aspects are detected in the CST systems currently existing in the building market, which are noted below: First, the solar thermal sector presents few systems capable of being architecturally integrated into buildings [23]. In part, this is due to the fact that most collectors have been conceived and designed using only efficiency parameters, without taking into account the location, climatic conditions, or architectural composition in which they will be installed. Some of the manufacturers even present an incorrect interpretation of the concept of integration, understanding it simply as an element that is flush with the rest of a building’s envelope, while architects understand that integration is a system that is capable of aesthetically inhibiting itself or that stands out with the same intensity as the rest of the components of the façade and/or roof. Second, most CST systems work based on the premise of the maximum performance; that is, they possess the minimum surface area and operate at high working temperatures. This concept is totally erroneous in certain climatic zones, such as the Mediterranean climatic zone, where solar radiation is abundant and constant throughout the year [24,25]. A system operating at high temperatures (between 90 and 120 °C) and/or medium-high temperatures (between 60 and 90 °C), in a climatic zone with a high solar radiation index, requires very high maintenance and control costs [26]. Third, and lastly, the final cost of commercialized CST systems is too high because of the excessive use of metal sheets and/or conduits or special coatings, which make the product excessively expensive, with a direct impact on the final amortization of the investment. For example, the main materials of the absorbers are generally copper, aluminum, and steel [27], and their respective special coatings are based on black nickel [28], black chrome [29], CoCuMnOx spinels [30], copper oxide [31], and carbon/nickel oxide [32], among others. Thus, solutions are required, where the cost does not penalize investors excessively [33].

It is important to note that because the entire exterior surface of a building is exposed to the sun, façades and/or roofs (including transparent and opaque parts) are susceptible to be energy-harvesting surfaces, thus maximizing the solar collection capacity [34] while reducing the performance of the collector itself. In conclusion, the large external surfaces of buildings compensate for the inherent intermittency and low performance (at working temperatures below 60 °C) that new CSTs may have [35,36].

Therefore, it is necessary to design new CST products that offer the optimal degree of integration [37], that are consistent, in terms of energy performance, with those climatic zones that present high solar radiation rates, such as the Mediterranean climatic zone, and that their final cost is affordable for as many people as possible [38].

There are a few commercialized systems without glass covers that work at low temperatures, such as the Capteur AS system by Energie Solaire [39] or the systems developed by the research team led by Dr. Luís Eduardo Juanicó [40,41]. Although these systems are designed to be installed on large outdoor surfaces and are, therefore, low-power systems, they do not have a sufficient level of architectural integration. In this sense, a clear example of good architectural integration can be found in the Thermoslate product by the Cupa Group [42], which completely hides the collector part of the CST under a finish of slate stone tiles. However, the cost of this product is too high, and it does not offer the architect the possibility to play with the composition because it is only available in the traditional finish of dark gray flat slate. Other systems on the market, such as Techo Solar from SolTech Energy [43] and Solar Force from Eternit [44], pursue the same objective but with certain limitations because in both cases, the final finish is a glazed surface, and this does not allow complete integration with the rest of the envelope of the façade and/or roof.

In the ceramic materials sector, the advancement and appearance, in recent years, of new production technologies have led to considerable improvements in the technical and aesthetic properties of ceramics [45], achieving greater resistance to wear and high temperatures, greater thermal insulation capacity, lower density, greater hardness, and better anticorrosive properties. Among the most innovative production processes are the ceramic additive manufacturing technology, which eliminates mold requirements and offers the ability to produce complex structures [46,47], and the sintering process, based on a thermal process at high temperatures (above 1200 °C), which compacts fine particles of natural minerals, such as silica, quartz, and feldspar, and clays without melting them completely to provide the ceramic with greater mechanical strength, thermal stability, chemical resistance, and very low porosity [48]. These properties give ceramics significant value for industrial core components. Therefore, this work aims to discover the potential of ceramics to be used in the configuration of a new CST system and, thus, replace the metals and respective selective coatings existing in conventional CSTs currently marketed in the building sector. Previous research has already demonstrated the possibility of configuring a fully ceramic solar collector [49,50] that is characterized by its long service life, its great integration capacity into the exterior envelopes of buildings [51,52], and its suitability for climates with high solar radiation levels [53,54].

Previous research [35,38] has validated both the capacity of the architectural integration of the system and the possibility of creating an enclosure with dual functionalities: energetic and constructive, leaving in a more superficial analysis of the part related to the selection of adhesives, type of glass, thickness of the infrared trap, type of ceramic, internal configuration of the circuit through which the heat transfer fluid circulates, etc. For this reason, this second phase of research, presented in this article, has allowed us to analyze and study in depth all these aspects more related to the internal configuration and the behaviors of the different components in different situations of pressure, temperature, etc. In short, the following study provides answers to a set of essential questions for determining the collector’s performance, such as the selection of the type of ceramic, the adhesive between ceramic–ceramic and ceramic–glass layers, the distance between the glass and the ceramic absorber plate (infrared trap), expansion and tightness tests, pressure resistance, and, finally, a simple characterization of the collector. The overall good performance and results obtained validate ceramics as a configuring element of a new CST, although it is considered as necessary to advance further research that allows for greater energy efficiency without the need to install glass in front of the collector because this considerably limits the capacity for architectural integration.

A test campaign is presented below that provides an initial approximation and assessment of the technical and energy feasibilities of the new CST system made with ceramic materials.

From this introduction, in which the context of the study has been provided and the problem and motivation of the research have been defined, the article is organized as follows: Section 2 discusses the materials used (ceramic, glass, and adhesive) and the basic experimental methodology used to determine some determinant aspects in the configuration of the new CST. Section 3 presents the results obtained in tests related to the behavior of the materials in terms of expansion and watertightness, resistance to water pressure, and the energetic characterization of the CST. Finally, Section 4 summarizes the main conclusions, and Section 5 suggests future research directions.

## 2. Materials and Methods

First of all, the possibility of designing a ceramic collector without a glass cover (no infrared trap) has been studied, with the aim of eliminating the glazed and shiny appearance of conventional collectors. Bringing ceramics to the surface means obtaining a more architecturally integrated solution.

**Figure 1:**
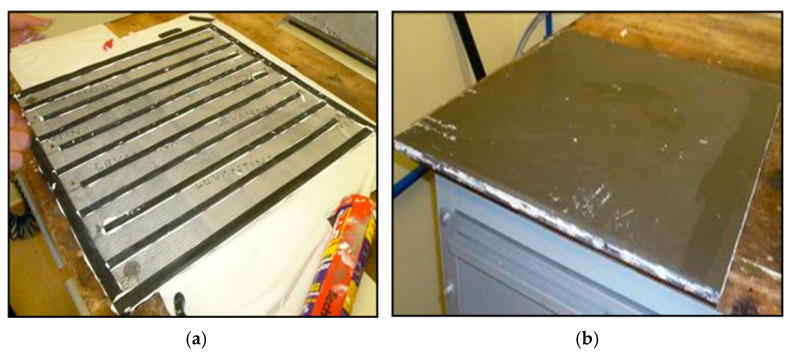
Images of the ceramic prototype assembly. (**a**) This image shows the interior part of the collector with the ceramic circuit walls; (**b**) this image shows the collector already closed with a ceramic panel.

To evaluate the performance of a collector with these characteristics, 60 × 60 cm prototypes were defined and built using 3 mm thick porcelain ceramic plates. Starting from the premise of not using metallic elements in the configuration of the new collector, it was decided to generate an internal circuit, between ceramic plates, in the form of a coil with a rectangular section through which the heat transfer liquid will circulate. This coil is generated by placing 9 mm thick ceramic bars and has an entry point and an exit point for the liquid (Figure 1).

Finally, a 40 mm thick extruded polystyrene thermal insulation plate was placed on the back of the prototype, and the entire perimeter of the prototype was sealed with aluminum film.

**Figure 2:**
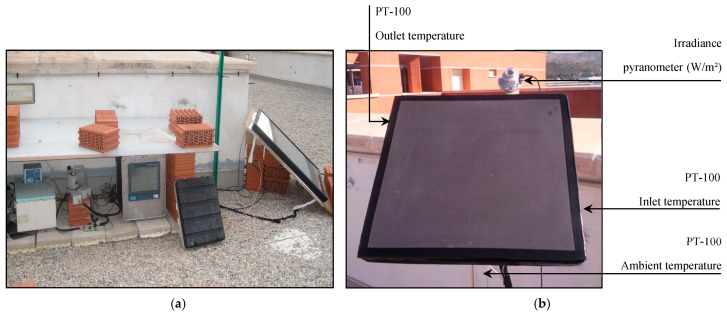
(**a**) Image of the experimental setup; (**b**) image of the ceramic prototype already finished and installed on the roof of the ITC laboratory.

Subsequently, tests were carried out at the facilities of the Institute of Ceramics Technology (ICT) in order to determine its performance and corresponding characteristic curve. For this purpose, the following experimental device has been used (Figure 2):

1. A data acquisition system: This collects the values of all the measured properties at a frequency of 1 min;
2. A thermostatically controlled bath: This device has a heating and a cooling system that keeps the water temperature constant above the ambient temperature;
3. A diaphragm pump: This is for the impulse of the water through the circuit in the thermostated collector bath;
4. A flow meter: It allows us to know the flow rate of the water circulating in the circuit (Q_v_);
5. Two four-wire Pt-100 resistance thermometers placed in the solar collector itself: They are used to measure the inlet and outlet water temperatures of the collector;
6. An overhead four-wire Pt-100 resistance thermometer placed at the bottom of the solar collector: This is used to measure the ambient temperature;
7. A thermopile pyranometer to measure the incident solar radiation (G_s_): It is installed in the same plane as the solar collector’s opening.

Finally, from the data collected during several days, the characteristic curve of the collector has been elaborated, which shows an excessively low efficiency, being well below the minimum efficiency of a commercial collector. It is, therefore, concluded that in terms of energy efficiency, the presence of glass and an air chamber in the form of an infrared trap is very important to reduce the losses produced by convection from the front face of the panel. For this reason, it was considered as necessary to carry out the same test by adding a 4 mm thick glass cover in front of the outer ceramic plate, generating an air chamber (infrared trap) of about 10 mm thick.

In the tests carried out on this last prototype, low yields were obtained, but sufficient to continue advancing in this line of research, considering the objective of achieving a low-yield system suitable for climates with high levels of solar radiation.

Subsequently, the objective is to further improve the performance of the collector by defining the different materials that make it up (glass, adhesives, thermal insulation, etc.) at the same time as studying their respective behaviors in relation to different service temperatures, differential expansions, and different pressures, among other aspects.

### 2.1. Ceramic Selection and Its Influence on Efficiency

**Figure 3:**
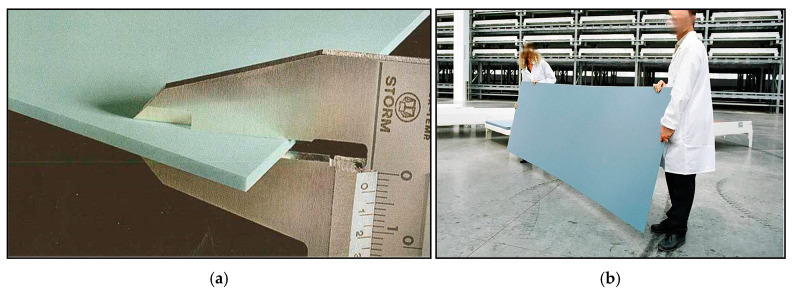
Images of the ceramic type used for the new solar collector. (**a**) Image showing that the thickness of the ceramic sheet is 3 mm. (**b**) Image showing the large size and format of this ceramic type (3200 × 1444 mm).

Ceramics play a fundamental role in the design of this system. For this, it is necessary to use a type of ceramic that is capable of adapting to the technical, architectural, and energy needs of the project. Currently available on the market is a type of large-format thin ceramic known as a large-format calibrated ceramic, which offers greater possibilities of finishes (formats, textures, colors, etc.) in addition to being a high-benefit product in terms of lightness, strength, flexibility, and thermal conductivity. These materials are manufactured using “TSP” technology (*Technology of Synthesized Particles*), a technological process of sintering and ultra-compaction that accelerates the metamorphic changes that natural stone undergoes when exposed to high pressures and temperatures for millennia. As a result, they are presented in large-format slabs (3200 × 1444 mm) and a wide variety of thicknesses from 3 to 30 mm (Figure 3). Because of their manufacturing process and intrinsic properties, they achieve a high level of performance, as shown in the parameters listed below (Table 1). These parameters are obtained from the manufacturer (Neolith) [55], one of the leading manufacturers in the production of this type of ceramic pieces. Among them, the following properties stand out: the radiation level (according to Equation (1)); high scratch resistance; low expansion coefficients; excellent fire resistance; resistances to ultraviolet (UV) rays, thermal shocks, and freeze–thaw cycles; and almost zero porosity (they have a water absorption of less than 0.1%), which offer a good performance in buildings’ envelopes. All these properties also allow vertical and horizontal joints between adjoining panels to be minimal, reaching a thickness of up to 3 mm.

The chemical composition of the product is totally inorganic and is based on three groups of elements: minerals from granite, minerals from glass and silica, and natural oxides. This raw material is wet ground and homogenized, after which inorganic pigments are added and dried to the appropriate particle size and humidity. After that, decoration systems are applied, and the forming is carried out, ultra-compacted at very high pressures. Finally, a thermal process is applied, resulting in large-format boards with a homogeneous body. According to their color composition, they can be classified into at least three families: light colors, medium colors, and dark colors. It is obvious that for this research, the dark colors will have better performances in terms of the absorbance and thermal conductivity.

In addition, this ceramic has very good technological properties in terms of resistances to frost, high temperatures, fire, UV radiation, bending, atmospheric agents, and corrosion; hardness (the hardness of the ceramic calibrated on the basis of the MOHS scale is equal to 8, and diamond has a value of 10); and lightness (21 kg/plate = 7 kg/m^2^).

The color of the ceramic has a significant influence on the energy efficiency of the collector. Absorbance (α) is the quotient of the absorbed solar radiation to the incident solar radiation according to Equation (1), based on the work of Duffie and Beckman [60]; therefore, its value varies between 0 and 1.

(1) $$\alpha =\frac{\mathrm{Absorbed}\;\mathrm{radiation}}{\mathrm{Incident}\;\mathrm{radiation}}$$

A body with an absorbance of α = 0 does not absorb any radiation. A body with α = 1 is a perfect black body because it absorbs all radiation. Real bodies have absorptivities between 0 and 1. The lowest values are around 0.03 (3%) for specular surfaces, and the highest values are 0.97 (97%) for matte black surfaces. In the particular case that the body is a flat surface, or can be assimilated to a more-or-less flat surface, when the solar radiation is incident, a part of it is absorbed, causing the heating of the same surface.

**Figure 4:**
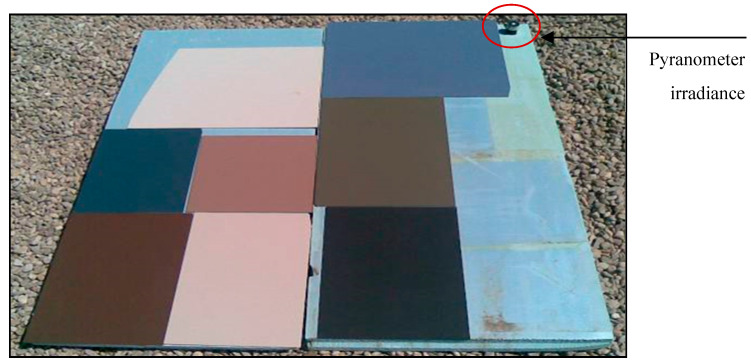
Samples of the different ceramics analyzed.

In order to estimate the absorption of the pieces used in the collectors, a simple test has been designed, consisting of placing pieces of different colors on a thermal insulation panel (extruded polyurethane that is 30 mm thick) so that the heat lost through the back of the pieces can be considered to be negligible. The colors of the pieces studied are the following (hereinafter, each piece will be identified by its color): black, green, dark blue, dark brown, light blue, light brown, beige, and white (Figure 4).

In the calculations, it will be assumed that the emissivity of all the parts takes a value of 0.9, regardless of their color. The emissivity is a dimensionless number that relates the capacity of a real object to radiate thermal energy with the capacity to radiate if it were a black body (A black body has a coefficient of *ε* = 1, whereas for a real object, *ε* always remains less than one) according to Equation (2) as follows, based on the work of DeWitt [61]:

(2) $$\varepsilon =\frac{\mathrm{radiation}\;\mathrm{emitted}\;\mathrm{by}\;a\;\mathrm{surface}}{\mathrm{radiation}\;\mathrm{emitted}\;\mathrm{by}\;\mathrm{the}\;\mathrm{same}\;\mathrm{surface}\;\mathrm{if}\;\mathrm{it}\;\mathrm{were}\;a\;\mathrm{black}\;\mathrm{body}}$$

Emissivity takes values between 0 and 1, and the higher it is, the greater the capacity of the body to exchange heat by radiation with its surroundings.

**Figure 5:**
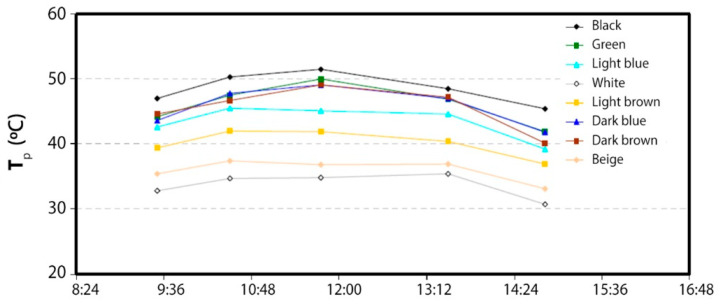
Evolution of the temperature of the parts during a sunny day.

The test consisted of recording the temperature of the parts (T_p_) exposed to solar radiation at various times during the day. The value of the ambient temperature (T_a_) and the value of the incident solar radiation (G_s_) were also recorded (Figure 5).

Applying an energy balance to one of the pieces, we have the following formula derived by Duffie and Beckman [60]:
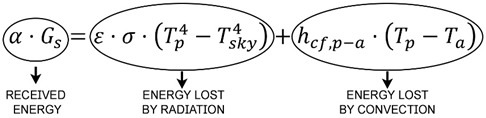
(3)
where σ is the Stefan–Boltzmann constant (5.67-10^−8^ W/(m^2^-K4);

T_sky_ is the temperature of the sky, estimated at T_a_-12 (K);

h_cf,p-a_ is the coefficient of the heat transmission because of the forced convection between the part and the environment (W/m^2^).

Because no absorptance value is available for any of the pieces, the results in this section have been expressed as the ratio between the absorptance value of the piece of a given color with respect to the absorptance of the corresponding black piece (α/α_black_), (Equations (6) and (7)). It should be noted that the absorptance of the black piece has been taken as a reference because it is the one that is likely to have a higher value. To obtain the results expressed in this way, the next equations, derived by Duffie and Beckman [60], for the energy balances of the piece of any color (Equation (4)) and of the corresponding black piece (Equation (5)) have been described.

Colored piece:

(4) $$\alpha \cdot G_{s}=\varepsilon \cdot \sigma \cdot \left(T_{p}^{4}-T_{sky}^{4}\right)+h_{cf,p-a}\cdot \left(T_{p}-T_{a}\right)$$

Black piece:

(5) $$\alpha_{black}\cdot G_{s}=\varepsilon \cdot \sigma \cdot \left(T_{p,black}^{4}-T_{sky}^{4}\right)+h_{cf,p-a}\cdot \left(T_{p,black}-T_{a}\right)$$

Dividing both equations, we have the following:

(6) $$\frac{\alpha \cdot G_{s}}{\alpha_{black}\cdot G_{s}}=\frac{\varepsilon \cdot \sigma \cdot \left(T_{p}^{4}-T_{sky}^{4}\right)+h_{cf,p-a}\cdot \left(T_{p}-T_{a}\right)}{\varepsilon \cdot \sigma \cdot \left(T_{p,black}^{4}-T_{sky}^{4}\right)+h_{cf,p-a}\cdot \left(T_{p,black}-T_{a}\right)}$$

Simplifying:

(7) $$\frac{\alpha }{\alpha_{black}}=\frac{\varepsilon \cdot \sigma \cdot \left(T_{p}^{4}-T_{sk}^{4}\right)+h_{cf,p-a}\cdot \left(T_{p}-T_{a}\right)}{\varepsilon \cdot \sigma \cdot \left(T_{p,black}^{4}-T_{skb}^{4}\right)+h_{cf,p-a}\cdot \left(T_{p,black}-T_{a}\right)}$$

**Figure 6:**
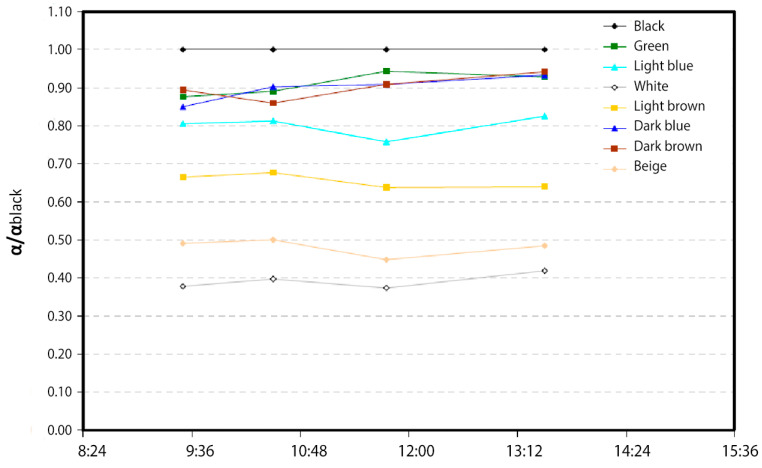
Evolution of the α/α_black_ ratio for the various parts over the course of a day.

For the heat transfer coefficient because of the forced convection between the parts and the environment (h_cf,p-a_), a value of 25 W/(m^2^-K) will be assumed. The evolution of the α/α_black_ ratio over the course of a sunny day is shown below (Figure 6). The fluctuations of this value for a given color are because of the experimental error in the measurement of the ambient temperature of the parts and because of the environmental conditions, mainly, wind, which vary slightly during data collection and directly influence the measured values. In any case, the dispersion of the data is quite reasonable.

The following table (Table 2) presents the average values of α/α_black_ for each of the pieces studied. First of all, it should be noted that there are three pieces’ colors (in relation to dark blue and brown), which absorptance values are not too far from the value of the corresponding black piece (0.90). In other words, these parts absorb 90% of the solar radiation compared to the energy that would be absorbed by the black part, which is a quite-favorable result when considering the manufacture of collectors with different colored parts. The values of the light blue and light brown parts are slightly lower (0.80 and 0.65 respectively), while those of the beige and white parts have much lower α/α_black_ ratio values (0.48 and 0.39). The test was carried out over several days, obtaining values equal to those presented below. Therefore, the use of parts with low values would not be at all favorable for the manufacture of collectors for water heating, but, on the other hand, their use could be interesting for the construction of collectors designed for cooling.

**Figure 7:**
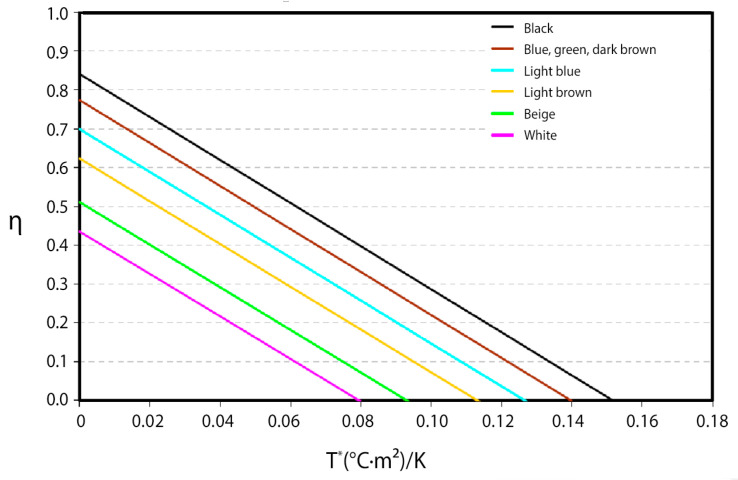
Theoretical characteristic curves according to the color of the ceramic pieces.

Subsequently, in order to have an idea of the effect of the color of the absorber on the collector’s performance, an absorptance of 0.9 (α_black_ = 0.90) was assigned to the black piece, which is a reasonable value, to, thus, obtain approximate values of the absorptance of the pieces of different colors. With these absorptance values, the different theoretical characteristic curves of the collectors built with each of the pieces of different colors have been obtained (Figure 7). This shows the enormous influence of the color of the absorber on the energy yield of the collector. It should also be noted that there are four colors that would be suitable for making collectors for water heating: black, green, blue, and dark brown. The blue and light brown colors would be in an intermediate situation and could be used as collectors in climatic zones with high solar radiation, as would be the case, for example, in the Mediterranean climatic zone. Finally, the beige and white colors, with much lower values than the rest, would be practically excluded in their function as collectors.

### 2.2. Definition of the Distance Between the Glass Plate and the Ceramic Absorber

First of all, the conditions for proposing the thickness of the air chamber, defined between the ceramic absorber plate and the glass cover, are studied. One of them is the heat transfer produced by the natural convection between flat and parallel plates.

The rate at which heat is transferred between two inclined plates at a certain angle above the horizon is of vital importance in the behaviors of flat plate collectors. The natural convection heat transfer coefficient is conventionally related to two or three dimensionless parameters: the Nusselt modulus (U_n_), the Rayleigh modulus (R_a_), and the Prandtl modulus (P_r_). Some authors relate the data in terms of the Grashof modulus, which is the quotient of the Prantl modulus to the Rayleigh modulus.

The Prantl, Rayleigh, and Nusselt moduli are determined according to the next equations [61]:

(8) $$Pr=\frac{\nu }{\alpha }\;Ra=\frac{g\cdot \beta^{’}\cdot \Delta T\cdot L^{3}}{\nu \cdot \alpha }\;Nu=\frac{h\cdot L}{k}$$

where h is the convective heat transfer coefficient;

L is the distance between the glass and the absorber;

K is the thermal conductivity;

G is the gravitational constant;

β’ is the coefficient of volumetric expansion (for an ideal gas, β’ = 1/T);

ΔT is the temperature difference between the plates;

V is the kinematic viscosity;

A is the thermal diffusivity.

For parallel plates, the Nusselt modulus is the ratio of the conduction resistance to the convection resistance (i.e., N_u_ = (L/k)/(1/h)), and a Nusselt modulus value of unity represents pure conduction. The ratio of the Nusselt modulus to the Rayleigh number for tilt angles between 0° and 75° can be calculated with good accuracy according to the following equation [61]:

(9) $$Nu=1+1.44\cdot [1-\left(1780\cdot \frac{sin{\left(1.8\cdot \beta \right)}^{1.6}}{Ra\cdot \cos\cos\beta }\right]\cdot {\left[1-\frac{1708}{Ra\cdot \cos\cos\beta }\right]}^{+}+{\left[{\left(\frac{Ra\cdot \cos\cos\beta }{5830}\right)}^{\frac{1}{3}}-1\right]}^{+}$$

where the meaning of the exponent “+” is that only the positive values of the terms inside the brackets are considered (zero is used if the terms are negative), and the meaning of *sin* is the sine function of the angle β (the inclination of the system with respect to the horizontal).

**Figure 8:**
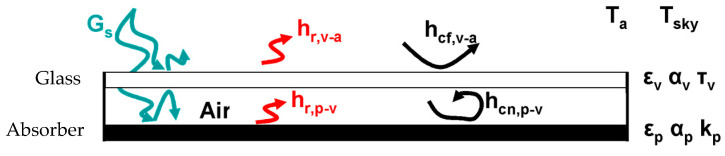
Schematic diagram of heat transfer phenomena between the absorber and the outside of the collector.

On the other hand, the heat transmission phenomenon between the ceramic absorber and the exterior is studied (Figure 8), detailing the incident solar radiation that reaches the collector (G_s_). A very small part of this radiation is reflected by the glass, another part is absorbed by it, and the most important part of it passes through the glass and reaches the absorber surface, which is heated. The glass is transparent to the radiation coming from the sun (short wave), while it does not let through the long wave radiation that is reflected by the absorber so that the glass cover acts as an infrared trap. Between the absorber and the glass, there is heat transfer by radiation and natural convection. Because of the low conductivity of the air, the heat transfer by conduction is practically negligible. Finally, the glass cover gives up heat to the outside by cooling because of the outside wind and by radiation. In this section the effect of the solar radiation will not be considered, but the calculations will be based on a given absorber temperature.

The heat transfer between the glass and the air because of the forced convection by the wind effect can be represented by the following equation [60]:

(10) $$q_{c,v-a}=h_{cf,v-a}\cdot (T_{v}-T_{a})$$

where q_c,v-a_ is the convective heat flux between the glass and the air;

h_cf,v-a_ is the heat transfer coefficient because of the forced convection (depending on the wind speed, a value of 15 W/(m^2^-K) is usually assigned);

T_v_ is the glass temperature;

T_a_ is the air temperature.

The radiative heat transfer between the glass and its envelope (the sky) can be represented by the following equation [60]:

(11) $$q_{r,v-a}=h_{r,v-a}\cdot (T_{v}-T_{a})$$

where q_r,v-a_ is the radiative heat flux between the glass and the air;

T_v_ is the glass temperature;

h_r,v-a_ is the radiation heat transfer coefficient, which can be calculated using the following equation [60]:

(12) $$h_{r,v-a}=\frac{\sigma \cdot (T_{v}+T_{sky})\cdot (T_{v}^{2}+T_{sky}^{2})}{\frac{1}{\varepsilon_{v}}+\frac{1}{\varepsilon_{p}}-1}$$

where σ is the Stefan–Boltzmann constant (5.67-10^−8^ W/(m^2^-K4);

ε_v_ is the glass’s emissivity;

ε_p_ is the emissivity of the absorber.

The heat transfer resistance between the absorber and the glass can be expressed as follows [60]:

(13) $$R_{p-v}=\frac{1}{h_{cn,p-v}+h_{r,p-v}}$$

Similarly, the resistance to the heat transmission between the glass and the exterior can be expressed as follows [60]:

(14) $$R_{v-a}=\frac{1}{h_{cf,v-a}+h_{r,v-a}}$$

Then, for this system, the top loss coefficient between the absorber and the outside (Us) can be expressed as follows [60]:

(15) $$U_{s}=\frac{1}{R_{p-v}+R_{v-a}}$$

**Figure 9:**
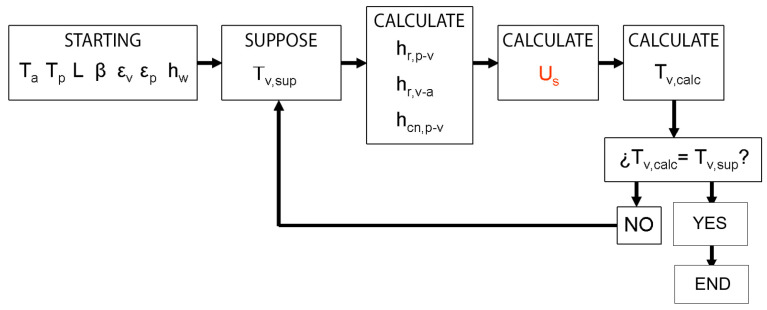
Schematic of the iterative process for obtaining U_sup_ for a given T_p_ value.

Next, the influence of the distance between the ceramic absorber and the glass (the thickness of the infrared trap) on the collector’s performance can be assessed. This influence has been analyzed by calculating the loss coefficient at the top of the collector (U_sup_). For this purpose, an iterative process (Figure 9) had to be applied for various absorber temperatures (T_p_), using the equations described in the previous section. This iterative process is based on making the first assumption of the glass temperature (T_v_), starting from some initial data, and the respective heat transfer calculations are performed. From these coefficients, the overall top loss coefficient (U_s_) is determined, which represents the amount of energy lost from the absorber to the outside through the glass cover. With this value (U_s_), a new value for the glass temperature (T_v,calc_) is estimated. If they do not match, i.e., there is a significant difference, the value of T_v,sup_ is updated with T_v,calc_, and the process is repeated until a value is found for which the difference between the two parameters is negligible. At this point, the process is terminated, and the value of T_v_ is adopted as the stable temperature of the glass. In this way, the procedure iterates until the cover’s temperature converges, ensuring an energy balance that is consistent with the heat transfer coefficients and the defined boundary conditions.

It should be noted that in the calculation of h_cn,p-v_, the average air temperature between the glass and the absorber has been considered as the air temperature. This air temperature is used to obtain the values of the thermal conductivity (k), thermal diffusivity (the thermal diffusivity measures the rate at which the temperature changes within a substance. In other words, it is the rate of change at which a material increases in temperature when it is brought into contact with a heat source) (α), and kinematic viscosity (the unit of the kinematic viscosity is the stoke or centistoke (1/100 of a stoke). The kinematic viscosity can be defined as the quotient of the absolute viscosity in centistokes divided by the specific gravity of a fluid, both at the same temperature) (v) of the air, which depend on this temperature. Once the value of U_s_ has been calculated, the value of the glass temperature can be recalculated from the following formula based on the work of Duffie and Beckman [60]:

(16) $$T_{v}=T_{p}-\frac{U_{s}\cdot (T_{p}-T_{a})}{h_{cn,p-v}+h_{r,p-v}}$$

This process is repeated until the temperature of the glass takes practically the same value between two successive iterations.

**Figure 10:**
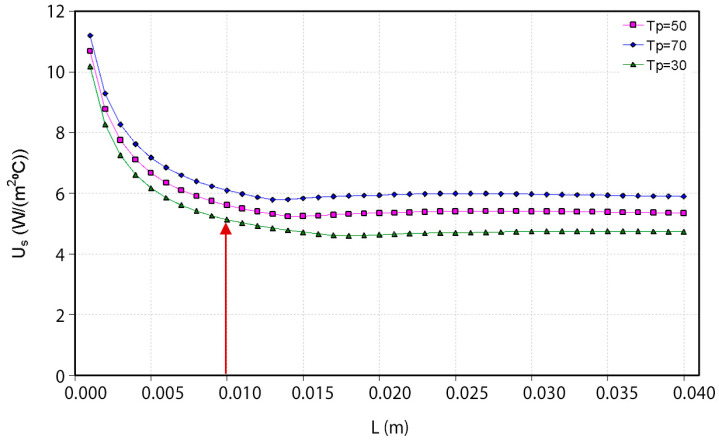
Evolution of the overall top loss coefficient of the collector with the absorber–glass distance (L).

The iterative calculation has been performed for distances between the ceramic absorber and the glass (L) from 0 to 40 mm, obtaining each temperature value for this distance and U_s_ value. Three absorber temperatures (T_p_) have been considered: 30 °C, 50 °C, and 70 °C. The results are shown in the following figure (Figure 10). For very short distances (from 0 to 4 mm), there is no convection, and heat transfer through the space between the glass and the absorber takes place by conduction and radiation. In this zone, the overall upper loss coefficient decreases rapidly as the distance between the glass and the absorber increases until it reaches the minimum at around 10–15 mm. When the fluid’s movement begins to contribute to the heat transfer process, the overall upper loss coefficient increases until it reaches the maximum at around 25 mm. A further increase in the distance between the glass and the absorber has hardly any influence on the overall upper loss coefficient. Therefore, it can be concluded that the appropriate distance between the glass cover and the absorber could be 10 mm because at this distance, the overall upper loss coefficient of the collector is minimal, and, consequently, the collector efficiency will be higher. It should also be noted that this value of the thickness of the air chamber is reasonable if it is considered that it is desired that the collector has a contained thickness.

### 2.3. Selection and Use of Adhesives

The objective of this section is to study different types of adhesives that allow the bonding of the ceramic base plate to the absorber and the absorber to the glass.

The selection has been made by taking as criteria the requirements demanded for each of the different joints to be made.

As mentioned in the final paragraph of the first section, previous research has already explored this topic. However, those studies were too superficial, prompting further investigation to determine more accurately the most suitable adhesive for the different joints within the solar collector.

For the bond between the glass and ceramics, the adhesive must offer high resistance to ultraviolet radiation to ensure its durability against solar exposure and environmental conditions. Additionally, it must withstand the shear stresses generated by the weight of the glass. Although the tensile stresses are relatively low, the adhesive must endure them continuously because of the difference between the thermal expansions of the glass and the ceramic absorber plate. Its primary function is to provide a secure bond that prevents moisture from entering the sealed air chamber between the materials.

On the other hand, the adhesive used for bonding the ceramic absorber to the ceramic base plate must be resistant to water and chlorine. This joint requires a higher tensile strength because it must withstand the pressure of the water circulating inside the internal cavity formed by the ceramic plates and the separating partitions. Furthermore, the adhesive must maintain its structural integrity within the operational temperature range of the solar collector, which can reach up to 100 °C. Unlike the previous adhesive, in this case, resistance to ultraviolet radiation is not necessary, as the joint is not directly exposed to sunlight.

The following companies specializing in adhesive materials have been contacted for this task: Sika [62], Quilosa [63], 3M [64], Bostik [65], and Bohle [66]. The company Bostik showed willingness to offer solutions for the two joints needed in the collector; however, after the initial conversations, we have tried to contact them again without obtaining any response. The company Bohle does not have any product in its catalog that meets the requirements to be used in the construction of the collector. The other three companies have been contacted, and their materials have been tested, with Quilosa being the company that has shown the greatest willingness to collaborate in this study.

First of all, it is decided to study the adhesive for the glass–ceramic absorber plate bonding. As mentioned above, the main requirement to be met by the selected adhesive material must be its resistance to ultraviolet radiation, and the service temperature of the adhesive must be up to 150 °C to have an acceptable safety margin of temperature resistance.

The *Sika* brand of adhesives offers various UV-resistant materials. There is a group of single-component sealing materials based on silane-terminated polymers (Silane or silicon (IV) hydride, which is a chemical compound with the formula SiH_4_), which are moisture curing. These materials have high resistances to ultraviolet radiation, as well as high tensile strengths, but the service temperature is between −40 °C and +70 °C, which is insufficient for their application as sealing materials between the glass and the absorber plate. The temperatures that can be reached by the absorber plate can easily exceed this value, especially in exceptional situations in which water does not circulate inside the collector and in which overheating of the collector would occur. Other adhesive materials based on neutral silicones also have excellent resistances to UV rays and aging. The main advantage is that the service temperatures of these materials are between −40 °C and +180 °C, and they also have good tensile strengths (although not as high as those of the previous group), which makes these materials candidates for use in the junction between the glass and the ceramic absorber plate. Materials of this type include *Sikasil WT-485*, which is a two-component silicone, and *Sikasil SG-20*, which is a highly elastic silicone. Both products are used in the construction industry for bonding and sealing glass façades.

For its part, the Quilosa brand recommends a product that can be applied for both glass–ceramic and ceramic–ceramic bonding. This product is a single-component structural silicone with neutral alkoxy crosslinking (in chemistry, an alkoxy group is an alkyl group bonded to an oxygen atom, i.e., RO-, where R is the alkyl group), and its trade name is *Orbasil Estructural*. This product is specially developed for its application in bonded-exterior-glazing façades. Because of its mechanical and application properties, it is suitable for overcoming the technical and safety requirements for its use in the assembly of glazing on a metal structure.

Finally, the 3M brand offered two bicomponent products, one epoxy based (*DP-190*) and the other polyurethane based (*DP-610*), which were not considered because of their high cost, as will be seen below.

With regard to the internal ceramic–ceramic collector junction, the adhesives commonly used in this type of junction have been analyzed. These are generally based on one-, two-, and three-component epoxy resins. Although these adhesive materials have very high tensile strengths, their main drawback is that their thermal resistances are not sufficient to preserve their properties within the temperature range to which they will be exposed. Another type of material that could be suitable is based on neutral silicone, which has high resistances to water and chlorine and a suitable service temperature range (from −40 °C to +180 °C). Also, the tensile strength of this type of adhesive, according to the manufacturers (1.5–1.7 N/mm^2^), is reasonably high and sufficient to withstand the pressures to which the joint between the two ceramic plates will be subjected, as discussed in the studies on the pressure resistance of the collector.

The Sika brand has a single-component polyurethane, which trade name is *Sikaflex-252*. The only drawback of this product is that its temperature limit for continuous operation is 90 °C, which is, perhaps, too low to be certain that this adhesive can withstand high temperatures. There are no other products that meet the water resistance requirement and have a higher temperature resistance.

Quilosa has two silicone-based adhesives, the aforementioned *Orbasil Estructural* and *Orbasil Energy*. The latter product is a sealant adhesive based on neutral crosslinking silicone rubber and is especially recommended for bonding and waterproofing solar panels and thermal collectors. The service temperature of this material is between −50 °C and +150 °C, and it has excellent water resistance.

**Figure 11:**
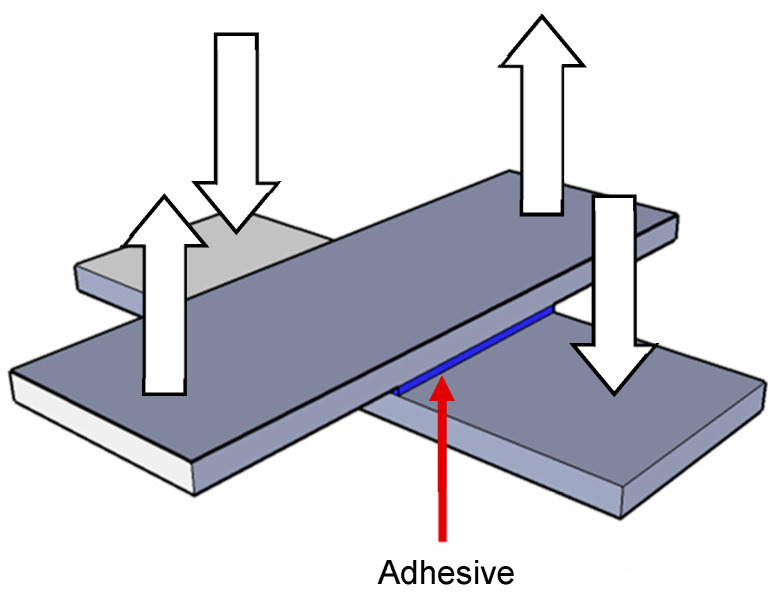
Schematic diagram of the setup for the determination of the tensile strength.

The solution provided by 3M is an acetoxy silicone sealant, known for its high heat resistance, withstanding temperatures of up to 260 °C under normal operating conditions. The adhesives supplied by these specialized companies were tested for tensile strength using a universal mechanical testing machine at ICT laboratories to evaluate their bonding performance with ceramic pieces. For this purpose, rectangular ceramic samples measuring 25 × 85 mm were cut and bonded crosswise with each of the adhesives analyzed (Figure 11), resulting in a bonding area of 25 × 25 mm. The samples were left to cure for three days to ensure complete adhesive polymerization. Before testing, the bonded specimens were immersed in a thermostatic boiling-water bath at 100 °C, subjecting the adhesive bond to high-temperature and high-humidity conditions to assess its performance under demanding environmental stresses.

Table 3 shows the tensile strength values of the adhesives tested (R_T, exp_). It should be noted that the *Sikasil WT-485* adhesive could not be tested because the supplying company did not provide any samples. The value of this property that appears on the technical data sheet (R_T, data sheet_) is 2.3 MPa, although it should be noted that this value would have been lower if it had been determined with the test conditions applied, as is the case with the corresponding values for the rest of the adhesives. The same table shows that the *Sikaflex-252* adhesive has the highest tensile strength. In fact, the experimentally determined value is three times that of any of the other materials tested. With respect to the other three adhesives tested, it should be noted that the two Quilosa adhesives have practically the same tensile strength value, with an error of around 10%, while the *3M structural* adhesive has a slightly higher value.

**Figure 12:**
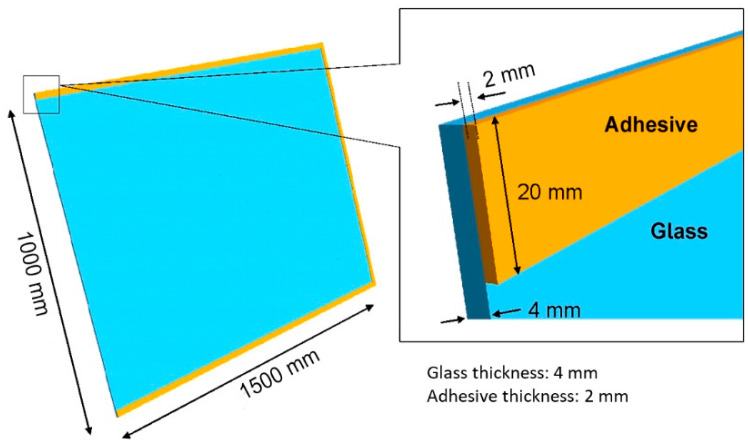
Geometry defined for the test, corresponding to half the system.

On the other hand, for the glass–ceramic joint, it is essential that the adhesive resists tensile stresses because of the differential expansion between the glass and the ceramic. Likewise, the adhesive used in this joint must support the weight of the glass placed on the absorber’s ceramic plate that is sealed perimetrically. In order to know the shear stresses to which the adhesive material will be subjected in this joint, a simulation model has been developed to perform this calculation. To do so, it has been considered that the adhesive layer has a thickness of 2 mm and a width of 20 mm (Figure 12).

The following table (Table 4) shows the values of the glass’s and adhesive’s properties in the simulation. A soft adhesive (Young’s modulus = 1 MPa) has been considered because the adhesives studied in this task present values of this property around this value.

**Figure 13:**
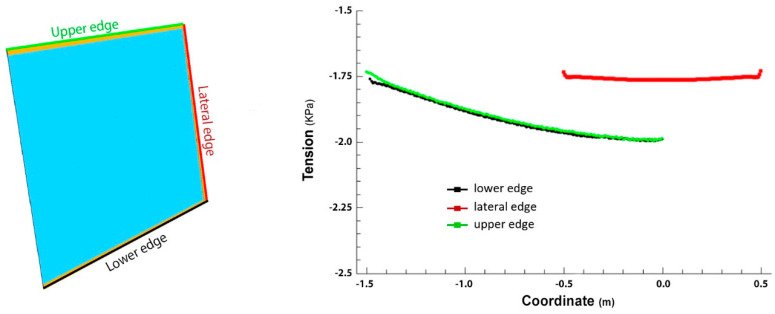
Shear computation zones and results.

The values of the shear stresses supported by the adhesive along each of the edges are shown below (Figure 13). It is noteworthy that the stress values are very low, on the order of 2 KPa. In fact, this value is less than 0.5% of the strength of the adhesive that has a lower value of this property (0.54 MPa), so the shear stresses generated at the edges of the perimeter sealing because of the weight of the glass (glass has a density of 2500 Kg/m^3^, which gives flat glass a weight of 2.5 Kg/m^2^ for each millimeter of thickness. Therefore, a 4 mm thick glass would weigh a total of approximately 10 kg/m^2^. In this case, the glass has a size of 1 × 1.5 m, so the weight of the element is exactly 15 kg), about 15 kg, do not pose any limitation on the choice of the adhesive material.

The last point to evaluate with respect to the choice of the adhesives studied is their cost. For this purpose, it was considered as appropriate to use the usual price list for these products, as shown in Table 5. Except for the *DP-190* and *DP-610* adhesives from *3M*, which have already been discarded from the beginning because of their high prices, the adhesive with the highest cost is the structural silicone from *3M*, probably because it is the one with the best thermal resistance. *Orbasil Structural* and *Sikaflex-252* have similar prices, while *Orbasil Energy* has a much lower price, taking into account that the two Quilosa adhesives have similar tensile strengths and that both are resistant to ultraviolet radiation. However, the favorable aspect for *Orbasil Structural* is that there is a lot of evidence supporting its durability during prolonged environmental exposure, with studies over more than 20 years according to the manufacturer. This gives us confidence in the applicability of this material for the bonding of the external glass of the collector, the placement of the latter on the façade, and the consequent safety aspects. As for the internal ceramic–ceramic bonding, any of the four adhesives can be used for that purpose, although *Sikaflex-252* would be questionable because its temperature limit for usage is +90 °C. Regarding the other three materials, the choice can be made by taking into account the economic aspect, with the *Orbasil Energy* adhesive being a good option, considering the performance/price ratio.

## 3. Results and Discussion

Once the section of the panels and the most suitable adhesives have been defined, relevant aspects, such as the airtightness of the collector when subjected to extreme conditions of pressure and temperature, begin to be analyzed in the laboratory.

In order to carry out these tests, small prototypes were built and tested to verify their physical integrity against the effect of the pressure and to verify that there were no leaks during their long-term operation.

### 3.1. Expansion and Tightness Tests

In addition to the pressure, the water circulation temperature has also been modified in the tests carried out to check that cracks do not appear as a result of thermal expansion.

**Figure 14:**
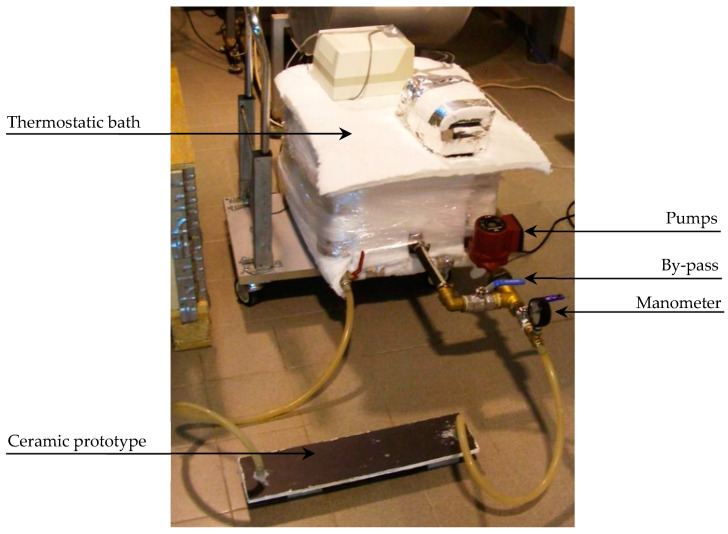
Experimental apparatus used for the tests.

To carry out this study, an experimental device was prepared (Figure 14) to regulate the pressure and temperature of the heat transfer fluid as it passes through the prototypes to be tested. This device consists of a thermostatic bath with one or two pumps installed at the outlet, allowing us to reach a fluid pressure of approximately 1 bar at the outlet. The use of one or two pumps depends on the pressure to be reached in the hydraulic circuit. The end of the outlet tube of the pumps is connected to the water inlet of the prototype to be tested. The heat transfer fluid then circulates through the prototype, the outlet of which is, again, connected to the thermostatic bath tank, thus creating a closed circuit. This has a recirculation or *bypass* branch so that the flow rate through this branch can be modified to regulate the fluid pressure in the other main branch of the circuit. In this branch, there is installed a pressure gauge that allows us to know at any time the fluid pressure as the fluid passes through the collector.

**Figure 15:**
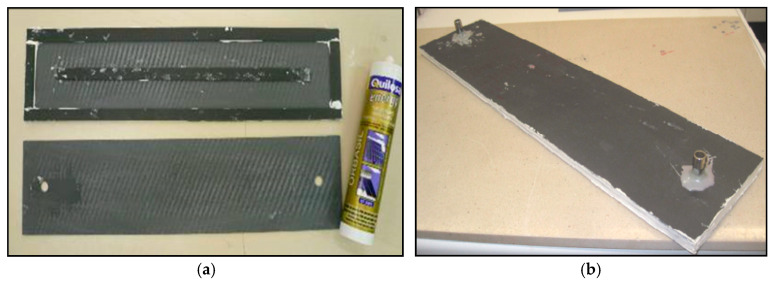
Images of one of the prototypes prepared for testing. (**a**) Interior view of the prototype, showing the cavity through which the heat transfer liquid will flow. The adhesive used for the ceramic–ceramic bond can also be seen. (**b**)View of the prototype once assembled, showing the two points of entry and exit of the heat transfer liquid.

Two prototypes were built for the tests, each with a different adhesive: *Sikaflex-252* (Sika) and *Orbasil Energy* (Quilosa), in order to study the integrity of the collector subjected to watertightness and expansion tests. The following figure (Figure 15) shows a view of the internal geometry of the prototypes and an image of one of them.

The design has been conceived to study the durability without having to prepare a full-size collector. The prototype has dimensions of 115 × 500 mm and consists of two channels arranged along its longest side, which are separated by a central partition of the ceramic material that is 15 mm wide. The prototype is sealed perimetrically by means of spacers that are 15 mm wide, also made of the ceramic material joined with the adhesive material to the upper and lower plates. The lower plate has two holes drilled into the base to allow the water to enter and exit this cavity by means of two dowels so that the cavity’s placement in the experimental setup can be carried out quickly and easily.

The pressure and temperature conditions to which the prototypes were subjected are presented in Table 6, according to the order in which they were applied. Each test was carried out for one hour under the specified conditions. Initially, the least severe conditions were tested, and the pressure and temperature values were progressively increased (in tests 1–5). In test 6, a sheet of glass was placed over the prototype, separating the two materials by means of a 10 mm air chamber, as it is arranged in the full-scale collector. The presence of the glass is intended to limit convective heat losses and make the temperature of the prototype higher than the case without the glass. This test was performed only at the highest temperature and pressure values. Additionally, in test 7, the water was replaced by a polyethylene-glycol-based antifreeze mixture (also known as polyethylene oxide (PEO) or polyoxyethylene (POE), polyethylene glycol (PEG) is a polyether widely used in industry. Polyethylene glycol is produced by the interaction of ethylene oxide with water, ethylene glycol, or ethylene glycol oligomers. The reaction is catalyzed by acidic or basic catalysts. It is preferred to start from ethylene glycol or its oligomers instead of water, as this yields low-polydispersity polymers) as the heat transfer fluid to test the behavior of a more viscous fluid than the first one.

The two prototypes withstood the heating–cooling cycles at different pressures to which they were subjected without any leakage or any other operating problem. Likewise, no cracks were observed because of thermal expansion.

### 3.2. Pressure Resistance of the Collector

In this section, the most suitable configurations to withstand the working pressures of the collector are analyzed, depending on the design and the thickness of the materials chosen.

The ANSYS 19 Mechanical program, finite element analysis (FEA) software, was utilized for this task to ascertain the stress distribution within the collector as a function of the following parameters:

○ the fluid pressure;
○ the fluid temperature;
○ the separation between the ceramic plates;
○ the width of the channels through which the water circulates (w);
○ the material properties (ceramic sheets, spacers, and adhesive).

In the initial calculations performed for spacings of 4 and 8 mm between the ceramic plates, it was observed that in this spacing range, the distance between the plates had no influence on the maximum principal stresses generated in the ceramic material and the adhesive.

**Figure 16:**
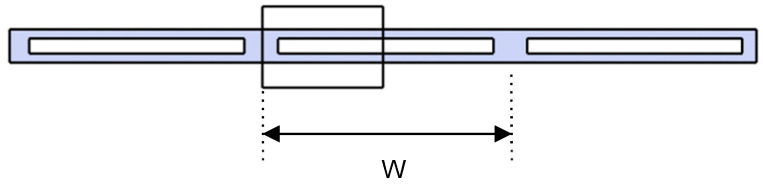
Analyzed section of the collector panel.

The section analyzed for the calculations is shown schematically below, which corresponds to a cross-section equivalent to half the fluid circulation channel (Figure 16). The material of the separator is the same as that of the ceramic plates and has a thickness of 4 mm. In the junction area, adhesive material with a thickness of 1 mm has been considered. Two adhesives with very different elastic properties have been studied: a soft one, with a modulus of elasticity of 1 MPa, and a rigid one, with a modulus of elasticity equal to 1 GPa. Regarding the distance between the centers of the spacers or the cavity width (w), calculations were performed for two fluid pressures: 0.1 and 0.2 bar.

**Figure 17:**
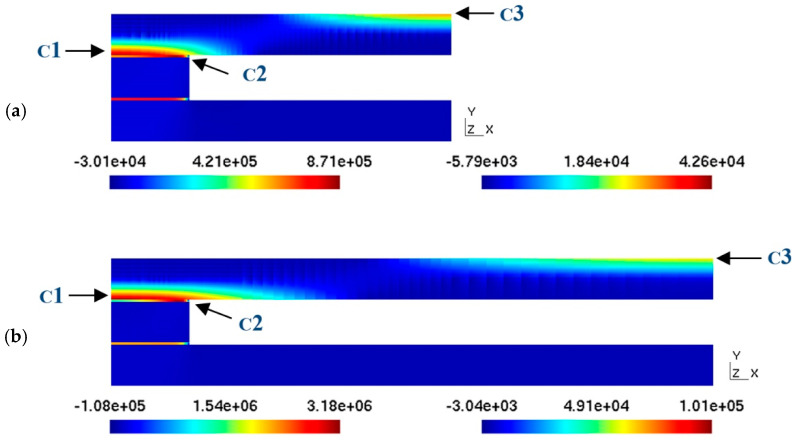
Stress distribution in the ceramic and the adhesive bond, considering a soft adhesive (E = 1 MPa) for two channel widths: (**a**) w = 50 and (**b**) 100 mm and a pressure equal to 0.1 bar.

The following figure (Figure 17) shows the distribution of the maximum principal stresses in the section considered for distances of 50 mm and 100 mm between the spacers (Figure 17a,b) in the case that the bonding material is a soft adhesive and the fluid pressure is 0.1 bar. This figure shows how there are three critical points to consider: point C1 corresponds to the central part of the junction of the upper plate with the separator; point C2 is located on the side of this same junction, and point C3 corresponds to the central part of the upper ceramic plate. At points C1 and C2, the stress can be calculated either on the ceramic side (cer) or on the adhesive side (adh); in each case, it will be specified where this stress is being determined. These three points are the ones that are subject to the greatest stress because of the curvature or buckling of the upper ceramic plate as a result of the fluid pressure inside the collector.

**Figure 18:**
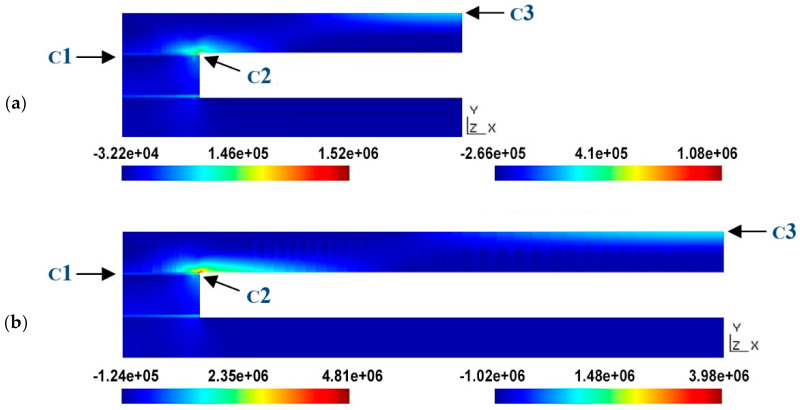
Stress distribution in the ceramic and adhesive bonding, considering a rigid adhesive (E = 1 GPa) for two channel widths: (**a**) w = 50 and (**b**) 100 mm and pressure equal to 0.1 bar.

In the case of a rigid adhesive (Figure 18), the only point with higher stresses is point C2-adh. In this case, all the stress is concentrated at this point because the adhesive hardly admits any deformation. At this point, the stress is on the order of 1 MPa for the case of a 450 mm cavity width, which would cause the adhesive material, which tensile strength takes values around 0.5 MPa, to break.

**Figure 19:**
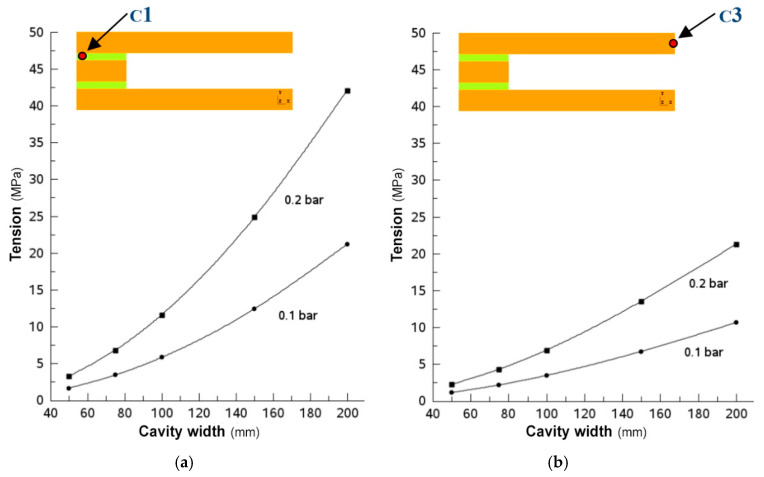
Maximum stress on the ceramic plate according to the channel width and pressure. (**a**) Stress at point C1-cer; (**b**) stress at point C3; soft adhesive, E = 1 MPa.

Figure 19 shows the principal stress in the ceramic, corresponding to points C1-cer (Figure 19a) and C3 (Figure 19b), as functions of the distance between the separators at the two pressures studied. From the graphs, it can be seen that the highest stress values are reached at point C1-cer. This is due to the fact that the curvature of the upper ceramic plate in the junction zone is greater than that in the central zone, and, therefore, the stresses in this junction zone are higher. In any case, the values for the case of the larger cavity width do not reach the value of the limit of rupture of the ceramic, which is on the order of 50 MPa, so the stresses supported by the ceramic would not cause its rupture within the range of pressures or the cavity width considered. It should be noted that the stress value is directly proportional to the pressure value, so if the pressure doubles, the stress value doubles as well.

In contrast to what was observed for the case of the ceramics, the maximum principal stress in the adhesive bond is critical because at a pressure of 0.2 bar, the value of the tensile strength (0.54 Mpa) of the most suitable adhesive material for this bond is exceeded.

**Figure 20:**
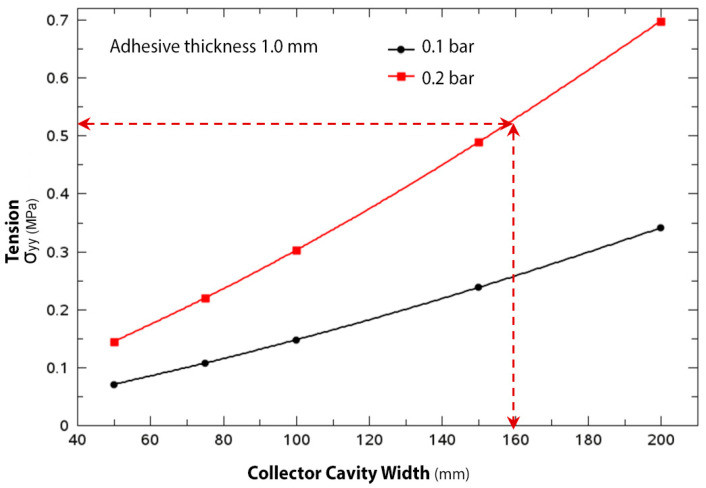
Stress (σ_yy_, the major component of the principal stress) in the adhesive bond as functions of the channel width and pressure. The stress at point C2-adh; (soft adhesive, E = 1 MPa).

Indeed, Figure 20 shows how, at a distance of 160 mm between the spacers, the principal stress in the adhesive bond reaches the breaking limit of the adhesive. In principle, the normal operating pressure of the collector is 0.1 bar, for which the width of the cavity could take higher values, probably around 300 mm. However, the design of the collector must be performed from a more conservative point of view, especially considering that the spacers’ spacing values considered are quite large. Therefore, the 0.2 bar curve has been used to define the maximum distance between the separators, which, in turn, will be reduced in the final design of the collector to a value of around 50 mm—considering a safety factor equal to 3—because at this value, the ratio between the area wetted by the water and the junction area will still be very favorable to heat transmission (50 mm over 4 mm), and the integrity of the collector should be affected by sudden increases in the pressure of the fluid inside it.

For the tests, the same assembly was used as that for the expansion and tightness tests, and the same ceramic prototypes were used, which were built with different adhesives: *Sikaflex-252* (*Sika*) and *Orbasil Energy* (*Quilosa*), in order to study the integrity of the collector subjected to the pressure test (Figure 14). For the prototype, an inner cavity width (w) of 50 mm has been determined, and the free passage area for the fluid is 35 mm.

Finally, during the performance of each test, it was verified that the prototypes did not suffer any type of alteration in this respect, withstanding perfectly the heating–cooling cycles at the different pressures to which they were subjected.

### 3.3. Characterization of the Ceramic Solar Collector

Once the most important variables for the configuration of the ceramic collector panel have been defined, the objective of this section is to assess its energy feasibility according to the UNE-EN 12975-2006 standard [63] applicable to solar thermal collectors. The final result will show the characteristic curve of the collector and a graph depicting the evolution of the difference between the outlet and inlet temperatures of the heat transfer fluid.

**Figure 21:**
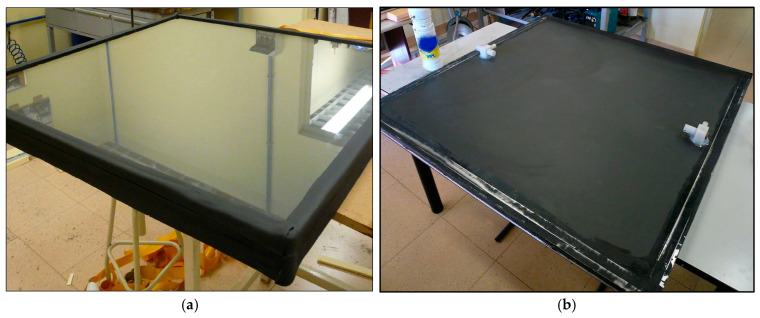
Prototype ceramic solar collector: (**a**) front face view; (**b**) rear face view.

To carry out the test, it was necessary to build a prototype of 1.2 × 1.2 m, with a parallel internal circuit model, a 4 mm thick glass layer to create an infrared trap (10 mm thick), and thermal insulation on its rear face (30 mm thick EPS). In Figure 21a, the front face of the prototype is shown, with the solar glass in front of the ceramic absorber plate, while in Figure 21b, the connection system through which the heat transfer fluid enters and exits is visible. For this, the same experimental device shown in Section 2 of this article was also used. The only additional element for this test was an anemometer, used to measure the wind speed.

**Figure 22:**
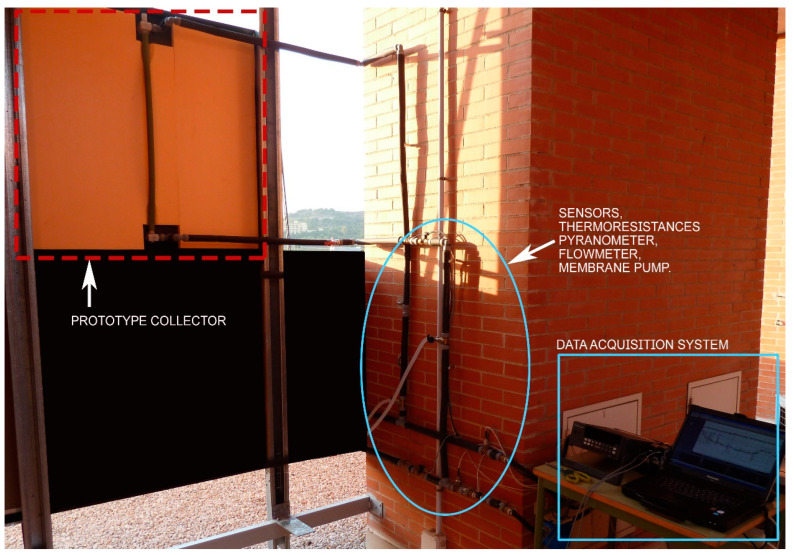
Image of the rear face of the test with the experimental setup used.

The rear face of the prototype collector is shown below (Figure 22), already built and placed at the test site (on the roof of the ICT laboratories), with the water inlet and outlet temperature sensors and incident solar radiation already installed on the laboratory roof. The heat transfer fluid circulates from the bottom to the top of the collector to avoid the formation of air pockets inside the collector, which would worsen its thermal performance.

The evolutions of some of the obtained parameters (ambient temperature, solar radiation, and inlet and outlet water temperatures) throughout a typical sunny day in February are of great interest and validate the good performance of the collector during moments of solar incidence on them.

The characteristic curve of the collector is an equation that relates its performance (η) to a parameter (T*), which is a function of the inlet water temperature (T_e_), the ambient temperature (T_a_), and the incident solar radiation (G_s_). Therefore, the determination of the characteristic curve consists of knowing the ratio η = η(T*).

The value of η is calculated as the quotient between the energy used to heat the water and the incident energy (solar radiation) measured directly with the pyranometer, according to the following formula [56]:

(17) $$\eta =\frac{Q_{V}\rho c_{p}(T_{s}-T_{e})}{G_{S}A}$$

where all the following parameters are known:

Q_V_ is the volumetric flow (m^3^/s);

ρ is the density of water (kg/m^3^);

c_p_ is the specific heat of water (J/kgK);

T_s_ is the water temperature at the outlet (K);

T_e_ is the water temperature at the inlet (K);

G_s_ is the solar radiation (W/m^2^);

A is the collector surface area (m^2^).

On the other hand, the T* parameter, also known as the reduced temperature, is calculated according to the following formula [56]:

(18) $$T*=\frac{T_{e}-T_{a}}{G_{s}}$$

The prototype has been installed at a 40° inclination above the horizontal. The main precautions taken into account, in accordance with the UNE-EN 12975-2006 [67] standard, include the following:

○ The hemispheric solar irradiance at the collector opening’s plane is greater than 600 W/m^2^ prior to the test;
○ The fluid flow rate is set at approximately 0.02 kilograms per second per square meter of the collector opening’s area and is kept constant throughout the test;
○ The test can only be performed if the wind velocity does not exceed 4 m/s.

To obtain the experimental data for the calculation of η and T*, the evolutions of the relevant parameters (T_a_, T_e_, T_s_, Q_v_, and G_s_) are recorded during several predominantly sunny days. In order to cover the whole range of the characteristic curve, different inlet temperatures of the circulating water are tested, corresponding to different values of the parameter T*. The test temperatures range from values close to the ambient temperature to values of around 85 °C.

**Figure 23:**
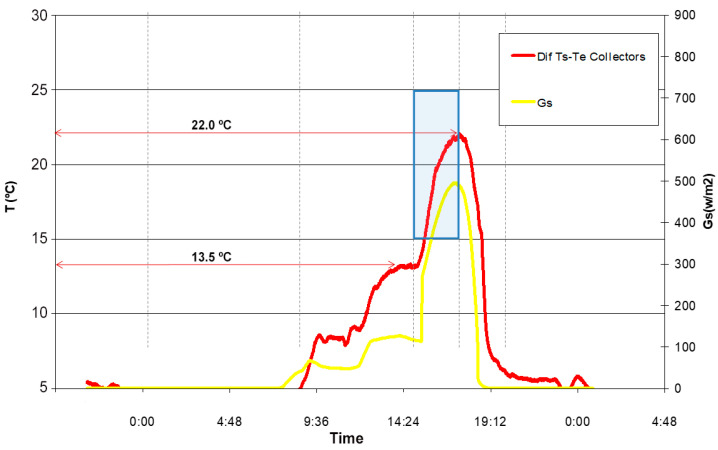
Evolution of the difference between the water’s temperatures at the outlet and inlet and the solar radiation intensity.

A more precise and clear way of explaining the heating effect of the heat transfer liquid in relation to the incident solar radiation is shown in the following image (Figure 23, obtained during a normal day in February (specifically, 22 February). A clear parallelism is observed between the temperature increase of the water as it flows through the collector and the intensity of the solar radiation. The average temperature increase reaches 13.5 °C at around 14:30 h, while by 17:00 h, it doubles, reaching a 22 °C difference. This, again, explains the good performance of the system when it receives direct solar radiation. One of the important bands in the following figure is the one shaded in blue. It shows how, initially, the two curves (between 13.5 and 15 °C range) are very close together (just at the moment when a shadow is cast over a large part of the collector panel because of the environmental conditions of the test). As the minutes pass and the sun’s rays are projected onto the panel again, the curves steadily and continuously move apart. This means that the increase in the temperature of the circulating water is more pronounced (the curve is more vertical) than the solar radiation index (W/m^2^).

Before presenting the characteristic curve of the tested ceramic collector, it should be noted that the following three parameters that provide information on the thermal behavior of a collector are extracted from the characteristic curve:

- The optical efficiency (η|_T*=0_): It is the value of the ordinate at the origin of the straight line and corresponds to the collector efficiency value when the T* parameter is equal to zero, i.e., the water inlet temperature is equal to the ambient temperature. This is the maximum efficiency that can be obtained with a collector. The higher it is, the better the thermal performance of the collector;
- The overall loss coefficient: It is the value of the slope of the characteristic curve and refers to the heat losses of the collector. The better insulated and designed a collector is, the lower the value of this parameter;
- The stagnation temperature (T*|_η=0_): This is the value of the parameter T* for which the collector efficiency is zero, i.e., the collector cannot raise the incoming water temperature for given ambient conditions. The higher the optical efficiency and/or the lower the overall loss coefficient is/are, the higher the value of this parameter.

Therefore, the energy efficiency of a collector will be higher the higher its optical efficiency and the lower its overall loss coefficient are.

**Figure 24:**
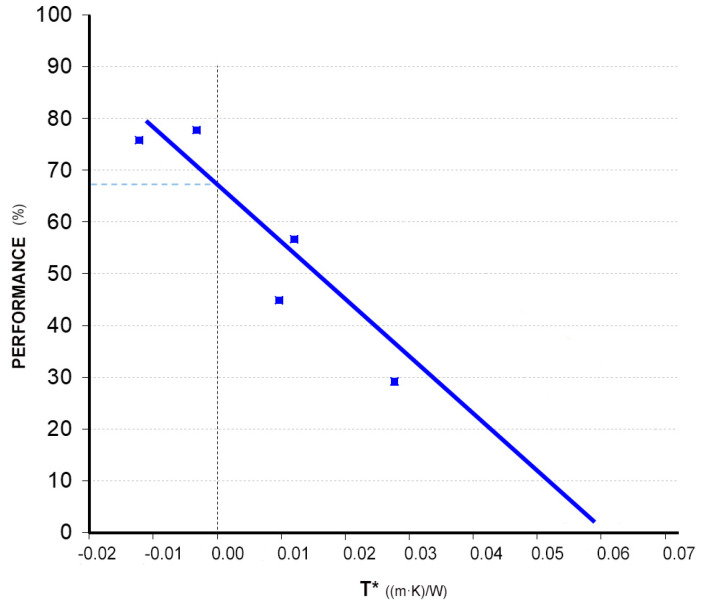
Characteristic curve of the ceramic collectors and experimental data measured at 30, 40, and 50 °C in February.

The characteristic curve of the tested ceramic collector is presented below (Figure 24), where each point on the graph corresponds to different inlet water temperatures. From this test, an optical efficiency value (η|_T*=0_) of 67.5% was obtained, with a global heat loss coefficient of 7.98 W/m^2^ °C and a stagnation temperature (T*|_η=0_) of 0.097 °C·m^2^/W.

It is, therefore, concluded that the obtained data confirm the collector’s low performance, although it remains optimal and sufficient if applied over a large surface area and in a region with a high solar radiation index, such as the Mediterranean climatic region.

## 4. Conclusions

As the presentation of the final model and the construction of the different prototypes have been completed, the conclusions and results obtained in the different sections are highlighted below:

- The first ceramic prototype without a glass cover has shown an excessively low performance so that to increase it, it is necessary to place a glass cover and its respective infrared trap in front of the collector. The tests carried out with the same prototype, but with a glass cover, continue to show a low performance, but they are sufficient, considering the low performance required in this project because of its suitability to the Mediterranean climate and large-surface application (roofs and façades);
- The influence of ceramics on the efficiency is first calculated by calculating the absorbances of various ceramic pieces of different colors. The values obtained confirm the possibility of using ceramics of darker colors, such as black, blue, green, and dark brown. Blue and light brown ceramics present an intermediate situation, being able to be used in areas of high solar radiation, and white and beige ceramics are totally discarded because of their excessively reduced performances;
- The optimal distance between the absorber plate and the glass (infrared trap) is defined as 10 mm;
- The adhesive selected from a total of six adhesives from three different manufacturers was *Orbasil Energy* from *Quilosa*. This adhesive, because of its good results in tests and its low cost, is the most suitable for bonding the different elements that make up the collector (ceramic–ceramic and glass–ceramic joints);
- The expansion and tightness tests verified that the prototypes, tested at different temperatures and pressures, did not show any leaks. Nor have cracks been observed because of thermal expansion;
- The pressure tests of the collector define a cavity width (the distance between separators, as measured from their axis through which the heat transfer fluid will circulate) of a 50 mm maximum (W = 50 mm);
- According to the thermal performance characterization of the ceramic collector, the results show that the characteristic curve falls within the typical range of those for commercial collectors (with 82% for the optimal commercial collector and 65% for the minimum commercial collector) and that its thermal performance is good.

## 5. Future Prospects

The research conducted has identified some unresolved issues or areas for improvement that could be further explored and expanded upon in future studies. Below are some of these research directions:

- Enhancing the integration of the collector’s envelope by eliminating the glass cover on the collector elements: This objective involves analyzing new material configurations and possibly incorporating new components to define a collector panel with a 100% ceramic exterior finish, without a glass cover, while maintaining or improving the achieved energy performance;
- Analyzing and assessing the final cost of the proposed system and its maintenance cost: This objective aims to develop a real production project for the system, bringing it as close as possible to actual manufacturing and installation conditions for the collector panel in a building’s envelope. This would provide a more accurate and realistic cost per square meter (including production and installation costs);
- Studying solutions for the increasing use of flat and walkable roofs in construction: It is necessary to explore the possibility of designing a ceramic collector that can be installed as a flooring system for a flat and accessible roof. This application would likely require modifications in the material configuration, fastening system, and connection methods.

## Figures and Tables

**Table 1:** Properties of the material and respective values obtained in tests by Neolith.

Basic Requirements	Standard	Essential Characteristics	UD
Flexural strength and breaking load	UNE EN ISO 10545-4 [56]	Mean flexural strength	≥45 N/mn^2^
		Breaking load	≥13,000 N
Water absorption, open porosity, and densities	UNE EN ISO 10545-3 [56]	Water absorption by boiling	0.10%
		Water absorption by vacuum	0.10%
		Apparent relative density	≥2.4 g/cm^3^
		Relative density	≥2.4 g/cm^3^
Impact resistance determination	UNE EN ISO 10545-5 [56]	Mean restitution coefficient	≥0.85
Linear thermal expansion determination	UNE EN ISO 10545-8 [56]	Expansion between 30 and 100 °C	≤6.5 × 10^−6^
Fire safety	(UE) 2016/364 [57] y EN 13501-1 [58]	Reaction to fire	Class A1
UV radiation stability	ISO 11341:2004 [59]	ΔΕ* < 1	≤5000 h

**Table 2:** Average absorptivity values (α/α_black_) for each of the ceramic pieces analyzed.

Color	Black	Green	Dark Blue	Dark Brown	Light Blue	Light Brown	Beige	White
**α/α**black	1.00	0.91	0.90	0.90	0.80	0.65	0.48	0.39

**Table 3:** Tensile strength values of the adhesives tested.

Brand	Trade Name	Type	R_T,tab_ (MPa)	R_T,exp_ (MPa)
Sika	Sikaflex-252	Single-component polyurethane	~4.00	1.71 ± 0.84
Quilosa	Orbasil Energy	Neutral silicone	1.70	0.54 ± 0.06
Quilosa	Orbasil Structural	Structural neutral silicone	2.36	0.56 ± 0.05
3M	SC 6151	Structural acetoxy silicone	-	0.63 ± 0.09

**Table 4:** Properties of the glass and the adhesive.

Material	Young’s Modulus (GPa)	Poisson’s Ratio	Density (kg/m^3^)
Glass	60	0.23	2500
Adhesive	0.001	0.48	1400

**Table 5:** Approximate prices of the adhesives.

Brand	Trade Name	Price (Euros per 330 mL Cartridge)
Sika	Sikaflex-252	13.00
Quilosa	Orbasil Energy	8.00
Quilosa	Orbasil Structural	12.00
3M	SC 6151	19.10
3M	DP-190	96.70
3M	DP-610	101.60

**Table 6:** Pressure and temperature conditions to which the adhesives have been submitted.

Assay	Pressure (bar)	Temperature (°C)	Heat Transfer Fluid	Glass
1	0.1	60	Water	No
2	0.1	95	Water	No
3	0.2	60	Water	No
4	0.3	95	Water	No
5	0.5	95	Water	No
6	0.5	95	Water	Yes

## Data Availability

No new data were created or analyzed in this study. Data sharing is not applicable to this article.
